# Detecting the colonization of ericoid mycorrhizal fungi in *Vaccinium uliginosum* using in situ polymerase chain reaction and green fluorescent protein

**DOI:** 10.1186/s13007-020-00645-x

**Published:** 2020-07-30

**Authors:** Hongyi Yang, Xingyu Zhao, Lili Li, Jie Zhang

**Affiliations:** 1 College of Life Sciences, Northeast Forestry University, Harbin, 150040 China; 2 Key Laboratory of Saline-alkali Vegetation Ecology Restoration (Northeast Forestry University), Ministry of Education, Harbin, 150040 China; 3 Institute of Forestry Science of Heilongjiang Province, Harbin, 150081 China

**Keywords:** Ericoid mycorrhizae, *Vaccinium uliginosum*, GFP, Colonization, In situ PCR

## Abstract

**Background:**

Ericoid mycorrhizal fungi (EMF) play important roles in mineral cycling and plant nutrient acquisition, and they increase plant survival in nutrient-poor environments. In this study, we detected the colonization of EMF using a green fluorescent protein (GFP) expression method and in situ PCR.

**Results:**

Genetic transformants of *Cryptosporiopsis ericae* and Sordariomycetes sp. expressing GFP were obtained via *Agrobacterium tumefaciens*-mediated transformation. GFP transformants were able to infect *Vaccinium uliginosum*, and their fluorescence was visible in the hair roots. Both in situ PCR and the GFP-expressing method indicated that EMF could colonize the hair roots of *V. uliginosum* 2 weeks after inoculation.

**Conclusions:**

This research represents the first attempt to detect ericoid mycorrhizal colonization using in situ PCR. A GFP-expressing method is an excellent system for detecting the colonization of EMF, but it is dependent on the successful transformation and expression of the *gfp* gene. In situ PCR and the GFP expression may be developed as new tools to study the interactions of EMF both with ericaceous plants and with the environment.

## Background

Ericaceous plant species are widely distributed globally and are mostly found as understory species in the cold areas of boreal forests [1, 2]. The hair roots of ericaceous plants are profusely colonized by a specific mycorrhiza fungi, known as ericoid mycorrhizal fungi (EMF). EMF play an important role in mineral cycling and plant nutrient acquisition [2], and thus EMF increase the capability of a plant to survive in nutrient-poor environments [3]. EMF are important for both cultivated and wild ericaceous plants growing in stressful environments [4].

A deeper understanding of the EMF colonization process would be beneficial for the utilization of EMF in the cultivation of ericaceous plants [5]. The detection of colonization is a starting point in understanding how this symbiosis is established and functions [6]. Over the past few decades, many studies have isolated a large number of fungi from the roots of ericaceous plants and have inoculated them into sterile micropropagated seedlings to detect the colonization of inoculated fungi and their ability to form ericoid mycorrhiza [1, 7]. Currently, increasing numbers of fungal taxa forming ericoid mycorrhizae have been identified [7]. Several techniques, such as trypan blue staining, electron microscopy, immunofluorescence, and in situ hybridization [8–10], have been developed and used extensively in a variety of biological tissues to evaluate microorganism colonization. Most previous studies used trypan blue staining to investigate EMF colonization [11, 12]. Several fungi, such as members of the *Phialocephalae*-*Acephala* complex, are considered as putative EMF based on the detection of fungal colonization by trypan blue staining [9]. However, the status of fungal colonization remains questionable, and some fungal colonization is likely to be the result of opportunistic colonization [1]. Recently, in situ PCR and fluorescent reporter proteins were also introduced to analyze microorganism colonization [13, 14]. In situ PCR, which combines the advantages of high-efficiency PCR amplification and the precise localization of in situ hybridization, could help elucidate microbial distributions and microbe–host interactions [15]. In addition, in situ PCR has the ability to detect and illustrate microorganism distributions in tissue sections for single-copy molecules and is thus advantageous for the investigation of microorganism colonization [16, 17]. Fluorescent reporter proteins are also essential for studying microbe–host interactions [18]. Green fluorescent protein (GFP) is one of the most common fluorescent reporter proteins, and it has been used to detect fungi *in planta* and observe fungal distribution and proliferation [18, 19]. GFP has been successfully expressed in several fungi and is used widely for *in planta* visualization [18, 19]. The majority of studies on mycorrhizal fungi have focused on the transformation of arbuscular mycorrhizae (AM) fungi, and several AM fungi have been documented [20]. In a study on EMF, Martino et al. [13] reported the successful and stable transformation of GFP using protoplasts and *Agrobacterium tumefaciens*-mediated transformation methods. In this study, we detected the colonization of EMF using in situ PCR and a GFP expression method. Both in situ PCR and GFP expression indicated that EMF could colonize the hair roots of *V. uliginosum* 2 weeks after inoculation.

## Results

### Observation of the fungal colonization of *V. uliginosum* by scanning electron microscopy

**Fig. 1:**
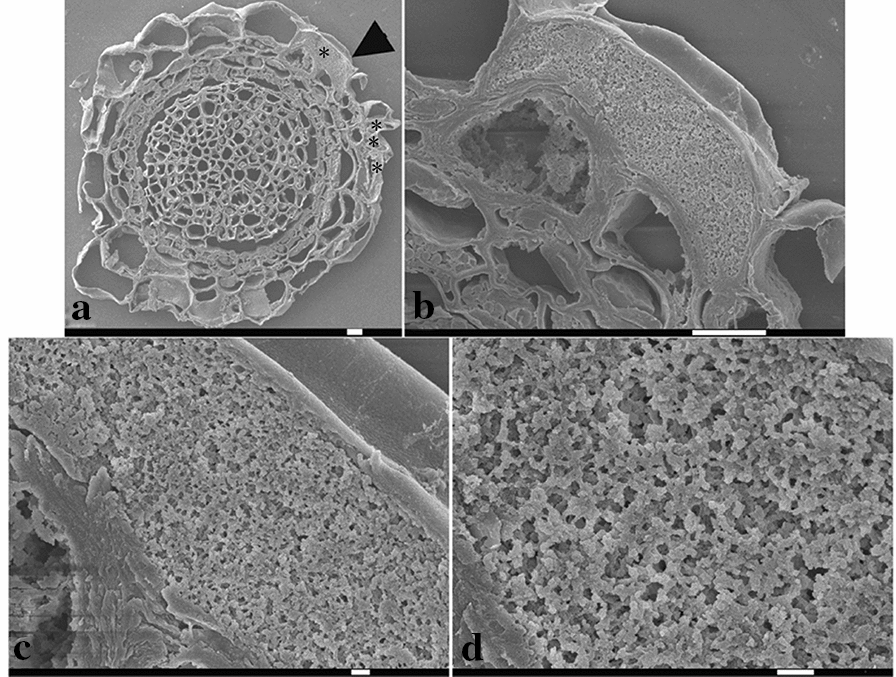
Observation of ericoid mycorrhizal roots from *V. uliginosum* by scanning electron microscopy. **a** Transverse sectional micrograph of an ericoid mycorrhizal root. EMF hyphal coils within the epidermal cells were labeled with asterisks. An epidermal cell was labeled with an arrowhead, and the magnified images for the cell are indicated in Figure **b**–**d**. Bars are 10 μm (**a**, **b**) and 1 μm (**c**, **d**)

Hair roots were collected from *V. uliginosum* growing in the Greater Khingan Mountains. Strain 103 of *Cryptosporiopsis ericae* was isolated from the hair roots of *V. uliginosum*. Micropropagated seedlings were inoculated with strain 103 of *C. ericae*. The fungal mycelia were observed to have colonized the hairs root by scanning electron microscopy. Several epidermal cells, which were located in the outermost cell layer of the hair roots, contained dense hyphal coils (Fig. 1). This feature was identical to that previously described for EMF [8]. Several epidermal cells were almost completely occupied by fungal hyphae (Fig. 1). Few fungal hyphae could be observed in the stele.

### Root colonization by fungal transformants expressing GFP

**Fig. 2:**
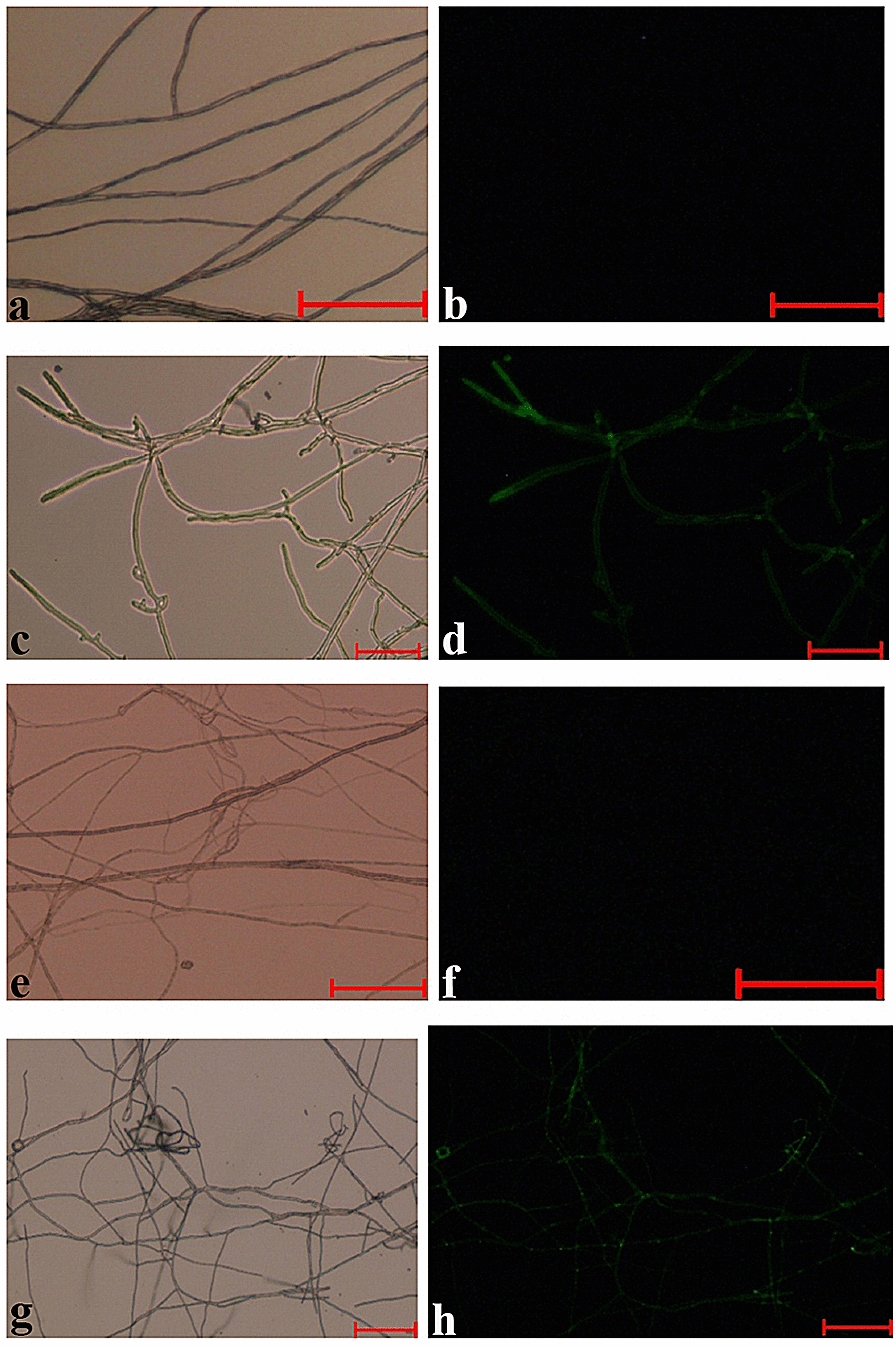
Microscopic images of *C. ericae* and Sordariomycetes sp. wild-type and GFP-transformed mycelia. No background signal is visible for the untransformed mycelia (**a**, **e** phase-contrast light images of *C. ericae* and Sordariomycetes sp.; **b**, **f** fluorescent light images of *C. ericae* and Sordariomycetes sp.). GFP expression in EMF is clearly visible in the hyphae as green fluorescence (**c**, **g**: phase-contrast light images of *C. ericae* and Sordariomycetes sp.; **d**, **h** fluorescent light images of *C. ericae* and Sordariomycetes sp.). All of the bars are 100 μm

Genetic transformants of strain 103 of *C. ericae* and strain 105 of Sordariomycetes sp. expressing the GFP were obtained via *A. tumefaciens*-mediated transformation. The specific GFP fragment was amplified using the total nucleic acids extracted from the hyphae as a template (data not shown). The fluorescence of the hyphae was observed by fluorescence microscopy (Fig. 2), and the fluorescence remained stable after repeated sub-culturing.

**Fig. 3:**
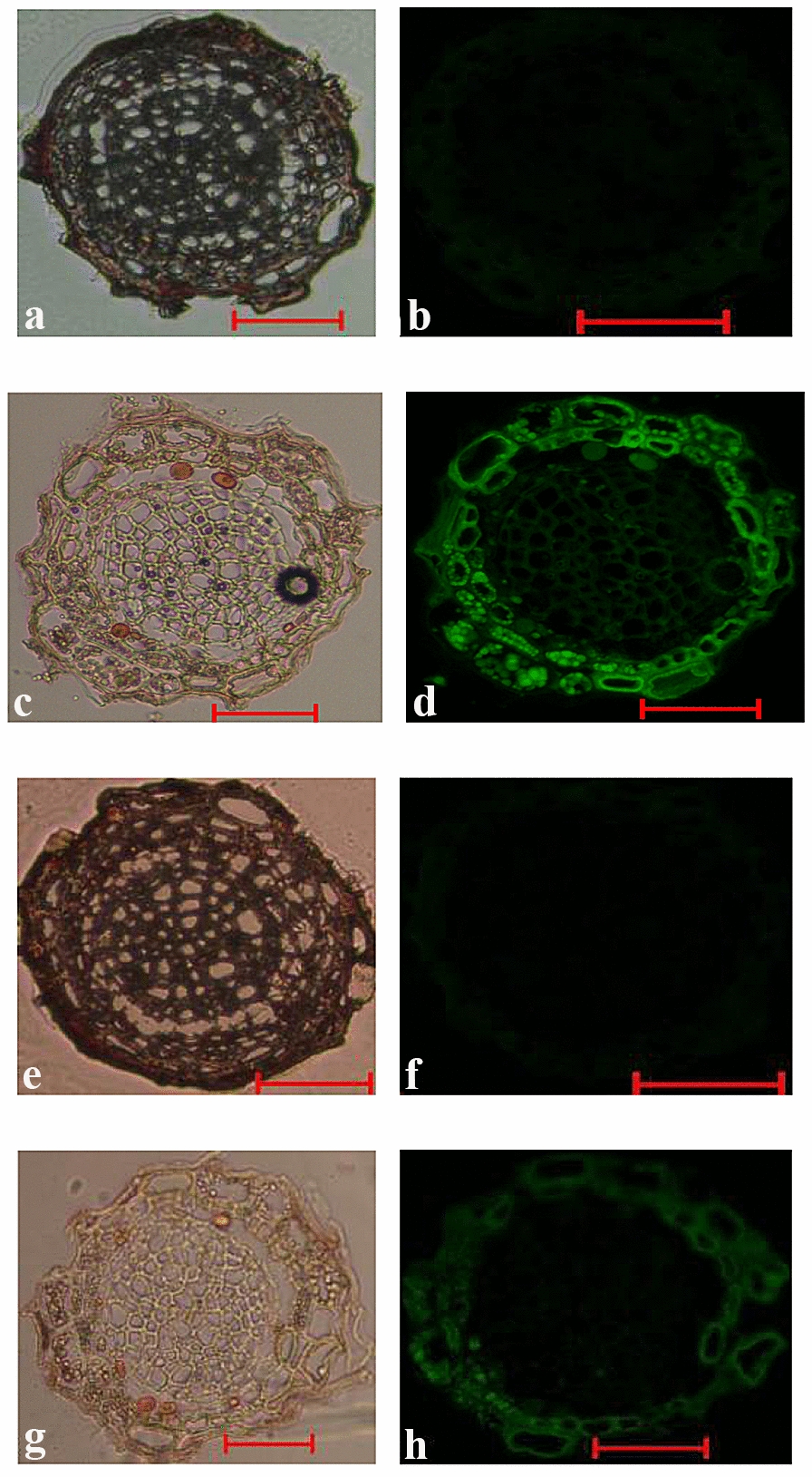
Microscopic images of *V. uliginosum* roots colonized by *C. ericae* and Sordariomycetes sp. wild-type (**a**, **b***C. ericae*; **e**, **f** Sordariomycetes sp.) and by *C. ericae* and Sordariomycetes sp. expressing GFP (**c**, **d***C. ericae*; **g**, **h** Sordariomycetes sp.). **a**, **c**, **e**, **g** phase-contrast images; **b**, **d**, **f**, **h** fluorescent images. Fluorescent fungal coils are visible in the root cells colonized by *C. ericae* and Sordariomycetes sp. expressing GFP at 2 weeks (**d**, **h**). Weak autofluorescence was observed in the root sections of *V. uliginosum* (**b**, **f**). All of the bars are 100 μm

The ability of *C. ericae* and Sordariomycetes sp. transformants expressing GFP to form mycorrhizae with *V. uliginosum* seedlings was determined via fluorescence microscopy. GFP transformants were able to infect *V. uliginosum*, and their GFP was visible in the hair roots, although *V. uliginosum* exhibited weak autofluorescence (Fig. 3). Infection of the axenic *V. uliginosum* seedlings with the GFP fungal mutants resulted in typical ericoid mycorrhizae, with hyphal coils that completely or partially occupied the root epidermal cells.

The colonization characteristics of EMF were also assessed. The EMF could form hyphal coils 2 weeks after inoculation, as observed by fluorescence microscopy (Fig. 3), suggesting that the EMF could invade the epidermal cells within 2 weeks and colonize the hair roots. Two weeks after inoculation, several epidermal cells of the *V. uliginosum* roots were infected by *C. ericae*, and larger hyphal coils could be observed; however, fewer epidermal cells were infected by Sordariomycetes sp., which formed smaller hyphal coils. This suggest that *C. ericae* has a more rapid invasion and hyphal coil-forming process than Sordariomycetes sp.

### Analysis of EMF colonization by in situ PCR

Total nucleic acids extracted from the fungal hyphae were used as the template. A digoxigenin-labeled DNA probe was prepared by PCR using the ITS1/ITS4 primer pair, and the predicted products of the labeled probe were obtained.

The probe was tested via hybridization to membrane-blotted nucleic acids according to standard methods, following which the hybridized probe was immunodetected with an anti-digoxigenin antibody and visualized with the colorimetric substrates nitro-blue tetrazolium chloride (NBT)/5-bromo-4-chloro-3′-indolyphosphate p-toluidine salt (BCIP). Based on the digoxigenin-labeled DNA probe and hybridization system in the membrane, we developed the in situ PCR system.

**Fig. 4:**
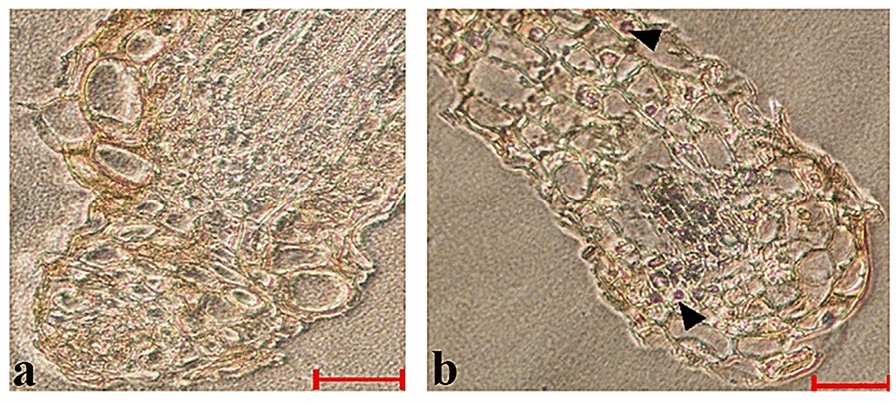
Analysis of EMF colonization by in situ PCR. Bar is 20 μm. **a** negative control; **b** longitudinal sections of the roots of *V. uliginosum* 2 weeks after inoculation of EMF, indicating colonization. Positive signals are labeled with arrowheads

Frozen sections were prepared from the micropropagated seedlings of *V. uliginosum* inoculated by EMF, and the fungal colonization of *V. uliginosum* roots was investigated by in situ PCR. The NBT/BCIP caused a redox reaction that formed an Alcian blue precipitate, which indicated the fungal location in the tissue. In general, there were several purple Alcian blue precipitate signals in the sections from the inoculated samples, whereas Alcian blue precipitate did not appear on the uninoculated samples (the negative controls) (Fig. 4). EMF could be detected efficiently in the sections of hair roots from *V. uliginosum* 2 weeks after inoculation. As observed in the GFP-expressing analysis, the distribution of positive signals was non-uniform.

## Discussion

Mycorrhizal formation involves a series of complex processes from constant mutual antagonisms to balance between fungus and host [21]. Most studies have focused on the colonization processes of AM fungi [22–24]. In contrast, there have been fewer studies on the processes of EMF colonization [8]. During the colonization processes of EMF, a symbiotic interaction may occur before actual fungus-root contact [25]. The metabolites, which are released into the rhizosphere, may trigger spore germination and regulate the tropism of the hyphae in the host tissues [26]. EMF must penetrate the cell wall of the host during the establishment of the symbiosis, and the process is likely to be enzyme-mediated [27]. In most cases, EMF hyphae penetrate the epidermal cell via a single penetration point [2]. In the present study, the invading EMF proliferated to produce numerous fungal hyphae, which occupied most of the volume of the plant cell (Fig. 1), finally, EMF formed a mature colonization. There have been fewer studies on the period from the earliest infection process to the establishment of the mature symbiosis between EMF and ericaceous plants [28]. Following inoculation of the hair roots of *Rhododendron ponticum* by EMF, colonization was detected in irradiated soil after 3 weeks [2]. Using in situ PCR and GFP expression, in the present study we found that EMF colonization could be detected in the hair roots of *V. uliginosum* 2 weeks after inoculation. Different fungi–host study systems may have important effects on the colonization time of EMF.

In situ PCR, which is based on PCR performed on fixed, whole cells, or sections, is a powerful tool for detecting the low-copy molecules [16]. In the in situ PCR system, fixed cells is the crucial reaction unit, which is considered as “semi-permeable amplifying bags” via proteinase K pretreatment [29], and fixed cells permit the entry of PCR components into the cytoplasm. The PCR product within the cell can be ultimately visualized by the color development system based on the labelled molecular-antibody-enzyme conjugate [17]. In situ PCR is often used in animal and medical studies, but fewer applications have been reported in plants [30, 31]. Currently, two main patterns, direct and indirect, have been developed for in situ PCR [31]. In the direct in situ PCR, some labeled nucleotides are directly incorporated into the PCR products, whereas for the indirect in situ PCR, the PCR products are detected by in situ hybridization using a labeled probe [32, 33]. In this study, we chose indirect in situ PCR, which is less convenient when there are more steps than the direct in situ PCR. However, indirect in situ PCR has good specificity and high amplification efficiency [34, 35]. In berry plants, secondary metabolites may have direct inhibitory effects on PCR amplification, and therefore the results of PCR were often inconsistent [36, 37]. The high sensitivity of the indirect in situ PCR may reduce the inhibitory effects on PCR amplification. In addition, the secondary metabolites are possibly swept out during the tissue preparation and pre-treatment processes. Generally, several conditions need to be optimized for new tissue when using in situ PCR due to different cell compositions, and the optimization process can be time-consuming [31, 32]. In addition, we used the universal primer pair ITS1/ITS4 to prepare the probe, and thus were unable to distinguish between the EMF and other fungi. The latter can be solved by designing a specific primer pair for a species of EMF.

This research represents the first attempt to detect ericoid mycorrhizal colonization using in situ PCR, and the EMF could be detected effectively in the sections by in situ PCR. Although many experimental procedures need be performed, in situ PCR is an excellent method for detecting EMF colonization.

Martino et al. [13] first reported an EMF transformant of *Oidiodendron* expressing the *gfp* gene. In this study, we reported the successful and stable transformation of two different ericoid mycorrhizal strains of *C. ericae* and Sordariomycetes sp. by *A. tumefaciens*-mediated transformation. Although the processes of transformation and expression of the *gfp* gene are laborious and time-consuming, the characterization of EMF colonization could be efficiently observed on the basis of a single operation using fluorescence microscopy in tissue sections without requiring cofactors or substrates. The main advantage of using fluorescent markers to detect EMF-plant associations over traditional techniques relies on the fact that fluorescent proteins do not require preparatory steps, which might affect the structure of the living cells [38]. As the GFP-tagged transformants maintain their mycorrhizal competence, it is more advantageous to study the early stages of symbiosis [13].

GFP tagging of a specific fungal strain depends on both the development of an efficient transformation protocol and the stable expression of the *gfp* gene in the fungus under natural environmental conditions [39]. However, unlike AM fungi (Glomeromycota), EMF are not monophyletic and contain functionally diverse groups [1, 2]. Therefore, for several specific EMF groups, it may be difficult to transform the *gfp* gene into the cells. In addition, the weak, interfering autofluorescence caused by the root tissues of *V. uliginosum* is undesirable. Application of laser scanning confocal microscopy is likely to partially avoid the autofluorescence of the root tissues [40]. In brief, the GFP-expressing method still constitutes an excellent system for detecting the colonization of EMF, but it is dependent on the successful transformation and expression of the *gfp* gene.

## Conclusions

We detected the colonization of EMF using a GFP expression method and in situ PCR in this study. Both in situ PCR and GFP expression indicated that EMF could colonize hair roots of *V. uliginosum* 2 weeks after inoculation. This research represents the first attempt to detect ericoid mycorrhizal colonization using in situ PCR. Both in situ PCR and GFP expression can be developed as new tools to study the interactions of EMF both with ericaceous plants and with the environment. Using specific primers corresponding to different fungi, the localization and proliferation characteristics of different fungi within hair root can be detected by in situ PCR. Similarly, the niche of different fungi can be analyzed using different fluorescent reporter proteins. Overall, in situ PCR and GFP expression will have important potential applications in mycorrhizal studies.

## Methods

### Plant materials and fungal strains

Root samples of *Vaccinium uliginosum* were collected from the Greater Khingan Mountains. To minimize damage to the roots, whole plants were removed with intact soil cores, placed into containers, and transported back to the laboratory. Strain 103 of *C. ericae* and strain 105 of Sordariomycetes sp. were isolated from the hair roots of *V. uliginosum*, and the strains were confirmed as EMF via resynthesis trials.

### Scanning electron microscopy

Hair root tissues of *V. uliginosum* inoculated with strain 103 of *C. ericae* were fixed with 2.5% glutaraldehyde for 6 h at 4 °C and sequentially dehydrated for 10 min in 30, 50, 70, and 90% ethanol solutions, and then twice for 20 min in 100% ethanol. The samples were freeze-dried and gold-coated by sputtering. Finally, the samples were observed with a field emission scanning electron microscope (JSM-7500F, JEOL Ltd., Japan).

### *Agrobacterium tumefaciens*-mediated transformation to obtain GFP-expressing fungi

**Fig. 5:**
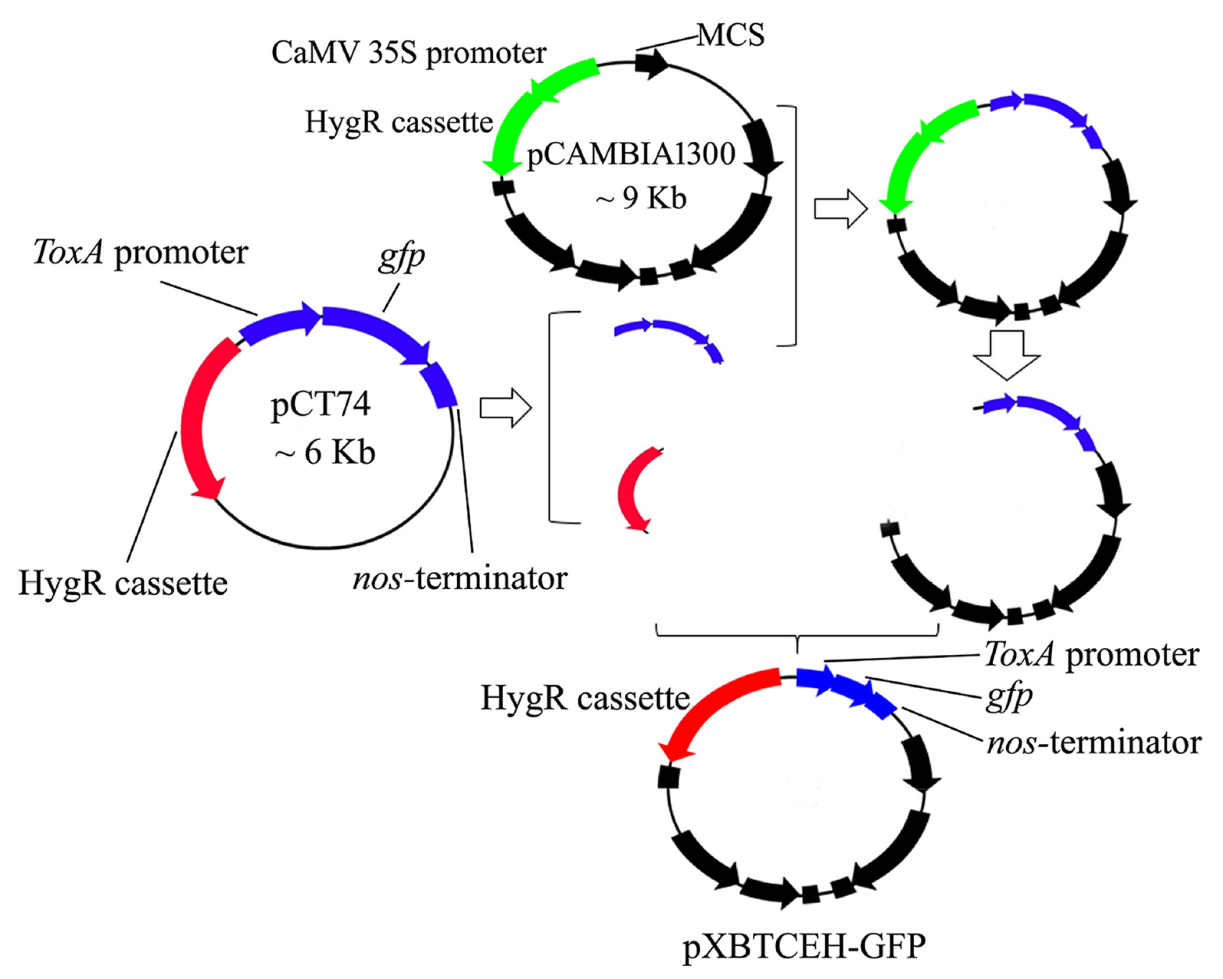
Schematic diagram for construction of the vector pXBTCEH-GFP based on the elements of vector pCT74 and pcAMBIA1300. MCS, multiple cloning site; HygR cassette, hygromycin resistance gene expression cassette

The plasmid vector pCT74, which contains the *gfp* gene driven by the *ToxA* promoter and hygromycin resistance gene expression cassette, was obtained from Bo Liu, Fujian Provincial Academy of Agricultural Sciences of China. The region containing the *ToxA* promoter, *gfp* gene, and *nos*-terminator of the vector pCT74 was amplified by PCR, and then the specific fragment was cloned into the multiple cloning site of the vector pCAMBIA1300. The cloned product was digested with restriction enzymes to remove the region of the hygromycin resistance gene expression cassette and CaMV 35S promoter. The digested product containing the pCAMBIA1300 backbone*, ToxA* promoter, *gfp* gene, and *nos*-terminator was ligated with the hygromycin resistance gene expression cassette of the vector pCT74, which was obtained by restriction enzyme digestion of the vector pCT74, ultimately yielding the binary vector pXBTCEH-GFP. PCR, molecular cloning, and digestion were conducted according to Sambrook and Russell [41]. A schematic diagram for the construction of the vector pXBTCEH-GFP is presented in Fig. 5.

Transformation was conducted as previously described by Martino et al. [13]. Briefly, *A. tumefaciens* strain EHA105/pXBTCEH-GFP was grown on YEP (yeast extract peptone) plates containing 50 µg/mL kanamycin. One colony was selected from the plate and grown in 100 mL LB liquid medium containing 50 µg/mL kanamycin at 25 °C 150 rpm for 21 h. The entire contents of the flask were collected and re-suspended in the induction medium containing 200 µM acetosyringone [13]. The suspension was then diluted to obtain an OD_600_ range from 0.15 to 0.20 and incubated at 25 °C and 130 rpm for 6 h (until OD_600_ reached 0.6–0.8). The mycelium suspension of strain 103 of *C. ericae* or strain 105 of Sordariomycetes sp. was prepared by filtering the mycelium from a shaking (120 rpm) culture grown at 25 °C. After 6 h, the bacterial suspension was mixed with the mycelial suspension (1:1, v/v). The content was evenly distributed onto Petri plates containing the co-cultivation medium and covered with sterilized cellulose dialysis membrane squares. Two days after plating, fungal transformants were selected by transferring the cellulose membranes to plates containing the selective medium (Czapek dox agar 1%) supplemented with 100 µg/mL hygromycin B and 200 µM cefotaxime, and incubated at 25 °C. Each transformant was subsequently transferred to Czapek dox medium containing 100 µg/mL hygromycin B.

The fungal transformants were further verified according to Silva et al. [42]. Total DNA from the liquid-grown mycelia of the transformants and wild-type strains was extracted using CTAB methods [43]. The *gfp* gene was detected by PCR using primers GFP-F (GACGTAAACGGCCACAAGTT) and GFP-R (CCTCCTTGAAGTCGATGCCC), which amplified a 330 bp sequence. Fluorescent emissions of the mycelia of putative transformants were examined with a fluorescence microscope (TE2000, Nikon, Japan) and images were recorded using NIS-Elements software (Nikon). The fluorescence stability of the transformants was detected by sub-culturing five times.

GFP expression by fungal transformants was also used in the *V. uliginosum* roots. Sterile micropropagated seedlings were transplanted into culture bottles containing the sterilized soil mixture (peat moss/vermiculite [1:1, v/v], which had been autoclaved twice for 40 min), and woody plant medium (Yutong, Shanghai, China), and then the inoculated transformants expressing GFP were added. The seedlings were maintained in a growth chamber under a 22 °C, 16 h/8 h day/night cycle, and irradiation of 70 µmol m^−2^ s^−1^. Infection and fluorescence patterns inside the roots were evaluated by microscopy.

### In situ PCR

Hair root tissues of *V. uliginosum* inoculated with EMF were embedded in optimum cutting temperature compounds (OTC) and then frozen for 30 s in liquid nitrogen. Sections (thickness of 10 µm) were cut from each OTC-embedded sample on a freezing rotary microtome (CM1900, Leica, Germany). The sections were then collected with a paintbrush on Superfrost plus microscope slides (Thermo Fisher Scientific, Waltham, USA). Fixation of the tissue, dehydration, treatment of proteinase, in situ PCR reaction, and detection of hybridization were conducted according to Yang et al. [14] with minor modifications. During the proteinase treatment, digestion was performed for 5 min at 37 °C using 6 µg/mL proteinase K. The digoxigenin-labeled reaction was performed using a DIG DNA Labeling and Detection Kit (Roche, Germany) with the primer pair ITS1/ITS4 according to the manufacturer’s instructions. The sections were then observed under a microscope (TE2000, Nikon, Japan).

## Data Availability

All data generated or analysed during this study are included in this published article.
